# The Development of a Paediatric Phoneme Discrimination Test for Arabic Phonemic Contrasts

**DOI:** 10.3390/audiolres11020014

**Published:** 2021-04-07

**Authors:** Hanin Rayes, Ghada Al-Malky, Deborah Vickers

**Affiliations:** 1 Department of Speech Hearing and Phonetic Sciences, Faculty of Brain Sciences, University College London, London WC1N 1PF, UK; dav1000@cam.ac.uk; 2 Ear Institute, Faculty of Brain Sciences, University College London, London WC1X 8EE, UK; g.al-malky@alumni.ucl.ac.uk; 3 Sound Lab, Cambridge Hearing Group, Department of Clinical Neurosciences, Clinical School, University of Cambridge, Cambridge CB2 0SZ, UK

**Keywords:** auditory perception, speech test, paediatric, phoneme discrimination

## Abstract

*Objective:* The aim of this project was to develop the Arabic CAPT (A-CAPT), a Standard Arabic version of the CHEAR auditory perception test (CAPT) that assesses consonant perception ability in children. *Method:* This closed-set test was evaluated with normal-hearing children aged 5 to 11 years. Development and validation of the speech materials were accomplished in two experimental phases. Twenty-six children participated in phase I, where the test materials were piloted to ensure that the selected words were age appropriate and that the form of Arabic used was familiar to the children. Sixteen children participated in phase II where test–retest reliability, age effects, and critical differences were measured. A computerized implementation was used to present stimuli and collect responses. Children selected one of four response options displayed on a screen for each trial. *Results:* Two lists of 32 words were developed with two levels of difficulty, easy and hard. Assessment of test–retest reliability for the final version of the lists showed a strong agreement. A within-subject ANOVA showed no significant difference between test and retest sessions. Performance improved with increasing age. Critical difference values were similar to the British English version of the CAPT. *Conclusions:* The A-CAPT is an appropriate speech perception test for assessing Arabic-speaking children as young as 5 years old. This test can reliably assess consonant perception ability and monitor changes over time or after an intervention.

## 1. Introduction

### 1.1. Selecting Appropriate Speech Materials in Arabic

Approximately 319 million people in the world speak Arabic, inclusive of all dialects and forms, making it one of the five most spoken languages worldwide [1,2]. Native-Arabic speakers are spread all over the globe as Arabs are amongst the fastest growing diaspora population in the world; they make up 4% of Berlin’s population in Germany, 4% of Belgium’s population, 2.5% of France’s population, nearly 1.5% of the United Kingdom’s population, and 1.1% of the United States’ population [1,2,3,4].

Similar to Modern Greek, Swiss German, and Haitian Creole, Arabic is a diglossic language where the same speaker uses different forms of the language in different settings [5]. There is a coexistence of two varieties of the language throughout the community, one of which is the literary or formal dialect while the other is a colloquial dialect that is spoken on everyday basis. Colloquial dialects are the true mother-tongue of native speakers of Arabic, and they vary slightly within regions and between different parts of the country, as well as between different Arabic-speaking countries where dialects may even be incomprehensible [6]. The Modern Standard Arabic is the common form of Arabic that can be understood across this diverse Arabic-speaking population.

The diglossia and range of dialects add complexity when developing speech assessment materials. Ideally an Arabic language speech test should be appropriate for all Arabic-speaking countries and appropriate for use in other countries with large populations of Arabic-speaking individuals (e.g., Belgium, Germany, France, UK, and US) migrated from different countries. This diversity and complexity should be considered when working with the Arabic language and could potentially be the reason why few validated Arabic speech assessment measures exist. Fortunately, a common practice across all Arabic-speaking countries is to use the Modern Standard Arabic in formal settings such as education and media. This across-language shared approach enables us to use Modern Standard Arabic when developing Arabic speech materials that are relevant to many countries. One example of the use of this approach is the work of Ashoor and Prochazka [7] who developed the Saudi Speech Recognition Threshold (SRT) test using Modern Standard Arabic. Modern Standard Arabic is unfamiliar to some pre-school children because this form of language is not used in everyday conversations. It is the language formally studied in schools, used in the media, in children’s programmes and cartoons. Therefore, it is appropriate to use Modern Standard Arabic with children aged five years and older, because they attend schools and have had years of exposure to media and children’s television.

### 1.2. Hearing Loss: Prevalence and Assessment

The World Health Organization (WHO) estimated the prevalence of hearing impairment ≥35 dB HL among children|(5–14 years) to be 1.7% worldwide and 0.9% in the Middle East and North Africa [8]. Saudi Arabia, where this study took place, has a higher prevalence of hearing loss than the world and the Middle East and North Africa combined, with an estimation of 13% for both conductive and sensorineural hearing impairments [9]. A large-scale epidemiological study was carried out between 1997 to 2000 and reported that the percentage of Saudi Arabian children with confirmed diagnosis of sensorineural hearing loss (SNHL) was almost as high as the global prevalence (1.5%) where hearing impairment ≥70 dB was estimated to be 0.7% [9,10]. Such a high prevalence has been associated with congenital conditions [11,12], childhood onset hearing loss [13], and attributed to the common practice of consanguineous marriage, which concentrates genes known to cause hearing impairment [11,14].

Inevitably, the incidence rates of hearing loss are increasing over time [15] and the number of paediatric candidates for hearing devices is growing. Such a high incidence rate has resulted in Saudi Arabia establishing the largest centre for cochlear implants (CIs) in the Middle East and in addition there is extensive use of hearing devices [16]. For appropriate monitoring of paediatric outcomes over time, well-validated measures of hearing ability are required. Currently, there is an extremely limited selection of tests that can be used to reliably assess speech perception in Saudi-Arabic-speaking children. A common practice in Saudi Arabia is to use Arabic translations of English tests or Arabic tests that have been developed in other Arabian countries to assess speech perception in Saudi Arabian children. Clearly, tests translated directly from English to Arabic without proper validation with Saudi Arabic-speaking children are unlikely to be balanced and the reliability poor. Similarly, tests that were developed in other Arabic countries often include unfamiliar words to Saudi Arabian children [17]. In the current practice, the reliability of speech tests used with Saudi Arabian children is seldom known.

Speech perception tests are the primary outcome measures used to assess speech development for children with CIs [18] and are essential tools for the assessment and management of hearing loss. There is value in developing and validating reliable speech perception tests that can assess and monitor auditory perception since good auditory discrimination skills are essential for the development of speech and language in children [19,20]. With tools for the assessment of the discrimination of speech cues to provide frequency-specific information with known reliability, it would help clinicians to verify the benefits of hearing devices or assess effectiveness of habilitation interventions.

### 1.3. CHEAR Auditory Perception Test

There are many published speech perception tests in English for children that assess speech recognition, auditory discrimination, and or monitor progress of speech and language skills. This Arabic auditory perception test was developed based on the English CHEAR Auditory Perception Test (CAPT) [21], which provides valuable frequency-specific information about the audibility and discrimination of speech cues from the pattern of phoneme confusions produced after a child completed the test. The CAPT was shown to be sensitive to hearing aid gain settings [22] and was used along with the McCormick Toy test [23] to derive UK cochlear implant candidacy criteria [24]. It is a phoneme discrimination test, which consists of monosyllabic words in form of consonant-vowel-consonant (CVC) or consonant-vowel-consonant-consonant (CVCC) that differ in either first or final phoneme. It is a closed-set test that uses four response alternatives on each trial. All the items in the test are real words that are familiar to children in the targeted age range.

The CAPT was validated and the critical difference for the measure was calculated. The critical difference is a measure that takes into account the reliability of the materials. It is a measure that can be used on an individual basis to compare performance in two listening conditions (e.g., with and without hearing devices) or different hearing devices fittings. The calculation of the critical difference provides a range of values for each individual’s scores that indicates a “true” difference, only if an individual’s score in the second condition falls outside the provided range would this be seen as a genuine change in scores. The critical differences for the original CAPT were calculated and were consistent with other speech tests, where the theoretical critical differences were somewhat smaller than the obtained critical differences for children, indicating that the children are less consistent across the test and retest sessions than predicted.

### 1.4. Rationale and Aim of This Research

There are a limited number of published speech tests that were evaluated with Arabic-speaking children in Saudi Arabia; a summary of these tests is listed in Table 1. The scarcity of tools to help assess consonant perception in Saudi-Arabic-speaking children was the main motivation of this study but with the intention of providing a measure that is also relevant and usable for other Arabic-speaking children. Therefore, the primary aim of this study was to develop the Arabic CAPT (A-CAPT), a closed-set phoneme discrimination test in Modern Standard Arabic that was developed based on the British English CAPT. A secondary aim of this research was to investigate whether a discrimination test in Modern Standard form of a language, in this case Arabic, can assess consonant perception in school-aged children.

This work outlines a procedure for producing a carefully translated version of a speech test in another language. The stages were to: (1) develop the materials based on knowledge of the vocabulary and contrastive words, (2) evaluate the stimuli and the response pictures with an expert panel, (3) pilot the initial version of the materials in a group setting, using electronic response voting, to understand whether all words are understood by the target population and derive final lists, (4) run test–retest reliability with target population using individual testing approach.

## 2. Materials and Methods

**Figure 1:**
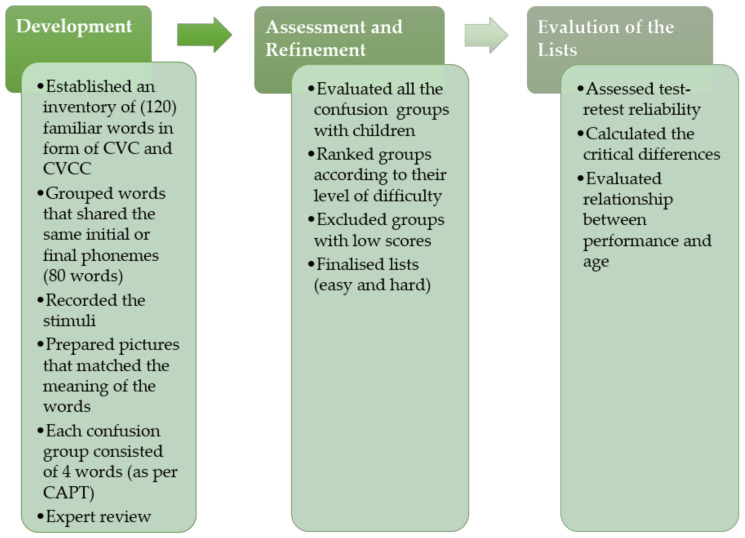
Summary of the process of development and evaluation of the test.

The process of the development and validation of this test was conducted in three main steps (Figure 1). The first step was the development of the materials; followed by the assessment and refinement of the test; and finally, the evaluation of the developed test.

### 2.1. Materials

To develop the A-CAPT, we established an inventory of 120 monosyllabic words found in Arabic children’s books and commonly used in everyday life that are familiar to Arabic-speaking children aged five years and older. We arranged the words in groups of four Arabic meaningful monosyllabic CVC or CVCC words that differ only in one phoneme. Words were grouped based on their consonant environments, where each selected word was in a group with three other confusable words. These confusion groups (CGs) containing the four similar words differed only in the first or final phoneme. The test therefore used a closed-set four-alternative-forced-choice test paradigm. Since not all the words in the inventory could fit into groups of four minimally contrastive words with similar phonemes, only 77 words were used and we produced 20 CGs consisting of 80 words, three words (/batˤ/, /tˤiːn/, /bar/, were presented in two CGs). Each CG contained four similar words that differed in either the initial (e.g., /jad/, /sad/, /yad/, /xad/) or final phoneme (e.g., /xatˤ/, /xas/, /xal/, /xad/). The groups were divided equally into two subgroups (ten CGs, i.e., 40 words each), one of which assessed the first phoneme and the other assessed the third or final phoneme. The subgroups were also divided equally into two subgroups for vowel length (five CGs). In total, there were two groups of words, first phoneme contrast and final phoneme contrast. Both groups contained forty words divided into twenty long vowel words and twenty short vowel words.

Familiarity and intelligibility of words within CGs was assessed by a simple binary forced response survey that was used with the three native Arabic audiologists (Saudi Arabian, Egyptian, and Libyan) who volunteered to assess the appropriateness of the materials for the target group of children. In a group setting, the clinicians listened to each stimulus and matched it to the corresponding picture then decided whether it was appropriate or inappropriate. The group agreed that the word /ɣaːb/, which refers to a verb form of the word ‘absent’, was rather abstract and the illustration may cause confusion to the children. Accordingly, the CG that contained this word was marked for elimination in the final version of the word lists. To keep an even number of CGs for the purpose of creating equal word lists, we opted to eliminate three more CGs that was determined to be least familiar to children and use it instead for a practice run. Otherwise, the expert panel agreed that the words selected were appropriate and matched the illustrations.

#### 2.1.1. Recording Words

Three native Arabic-speaking adults volunteered to record the words in Modern Standard Arabic, two females and one male (age range 35–46 year old); each word was recorded twice. The female speakers were both originally from the central region of Saudi Arabia (Al-Qassim), the first speaker was a post-graduate student while the other speaker was an elementary-school teacher. The male speaker was from the western region of Saudi Arabia (Makkah) and was a university lecturer. The speakers were seated a meter away from the microphone. The stimuli were recorded in an Anechoic Chamber (AC) at University College London with a Bruel and Kjaer 2231 Sound Level Meter fitted with a type 4190 condenser microphone. The signal was digitised with a Focusrite 2i2 USB sound card at a sample rate of 44,100 Hz. Six continuous wav files were recorded using ProRec 2.4 [26], recording software developed at UCL. Automatic separation before and after utterances and labelling for each word was achieved through ProRec after filtering the .wav files with a high pass filter to reduce gross fluctuations (using a MatLab script). Finally, the RMS values for all words was equated.

Three Arabic native speakers critically listened to the recorded words and gave their feedback on pronunciation, clarity of the recordings, accuracy of utterances in the Modern Standard Arabic and their preferred speaker out of the three. Using google forms, the evaluators individually listened and rated each word as clear or not clear. One of the evaluators who completed the forms was a post-graduate student in linguistics and her Ph.D. project involved investigation of dialects in Saudi Arabia, the other two evaluators were highly educated clinical audiologists in Saudi Arabia. The three evaluators voted for the same female speaker, and thus her voice was selected for the A-CAPT.

#### 2.1.2. Handling Test Materials and Illustrations

**Figure 2:**
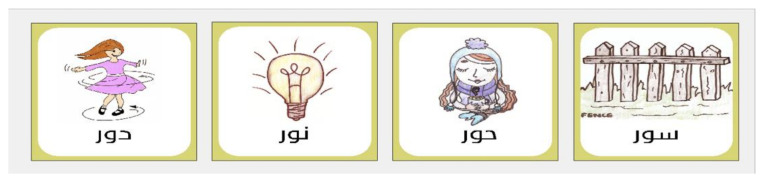
Example of a CG (/duːr/, /nuːr/, /ħuːr/, /suːr/) and their illustrations.

The pictures were all drawn by a 14-year-old child to ensure that they were relevant for younger children. Although there is no evidence that children’s drawings are necessarily more relevant to other children than professional illustrators, we chose to do this because in the original implementation of the CAPT some of the figures had to be altered to make them more meaningful for children. For example, the word peg was originally a picture of a clothes peg to be used on a washing line but had to be replaced with a clothes peg for hanging up coats. The pictures were then made into jpegs and a caption of the word written in Arabic was added to each figure (See Figure 2). After recording the words and matching them to corresponding pictures, the familiarity and appropriateness of the words and their corresponding pictures were assessed by an expert review panel, three Arabic-speaking audiologists, who listened and evaluated all the words and their corresponding pictures.

### 2.2. Methods

#### 2.2.1. Phase I: Assessment of Speech Materials and Development and Refinement of Word Lists

In this phase, we evaluated whether or not the selected words in Modern Standard Arabic language were appropriate and intelligible for the targeted age group of children.

##### Participants

Adverts were sent to the King Fahad Academy (KFA) in London, United Kingdom (UK) to recruit children for this experiment. The KFA is an independent school that follows the UK national curriculum and is funded by the Saudi Arabian Embassy in the UK. Twenty-eight children aged between six and eleven years (mean age = 8.94 years) were randomly selected from families that responded to adverts. All children were screened at 20 dBHL using pure-tone audiometry at frequencies 0.5, 1.0, 2.0 and 4.0 kHz. Transient evoked otoacoustic emission (TEOAE) was also performed on each child. All children passed the hearing screening and have no known learning disabilities.

##### Evaluation of the Test Material

Test materials, 80 monosyllabic words, were delivered via a computer using Prezi presentation slides on a screen through an EB-X62 EPSON projector. The words were presented at a soft presentation level calibrated to be 50 dBA at the centre of where the children were sitting for the testing to avoid ceiling effects. Words were delivered via a wall speaker Model NV-WA40W-SP to a group of normal hearing children. Tests were conducted in a classroom where the average background noise level of the room ranged 40–45 dB SPL; the noise level was measured three times within each session, before conducting the test, during the test and at the end of the test session. Each test was run twice with a ten-minute break between each session; the order of words was the same in test and retest sessions.

The classroom windows were shut to minimize external background noise. The classroom was allocated for non-English speaking children who receive extra language sessions and was located in the administration area which was generally the quietest in the school. No incidents of sudden background noise were observed during testing. The dimensions of the classroom were: 800 cm long, 704 cm wide, and 250 cm high. The children sat on the carpet in the middle of the room with the first row two meters from the loudspeaker. There were five rows of children in total and the calibration was conducted at the midpoint of these rows.

The children were instructed to select one out of four pictures that visually and orthographically represented the presented word. Children had a practice run that consisted of 4 words to familiarize them with the process. Following Vickers’ speech test procedure [27], each child was assigned a hand-held infrared transmitter to record her or his response to each trial. Turning point software and a USB receiver were used to capture the children’s responses. A rule from Vickers’ study was also followed, this was to exclude participants if they missed four or more items to rule out technical issues such as malfunction of transmitters, accordingly two children were excluded.

#### 2.2.2. Phase II: Evaluation of the Developed Lists

**Figure 3:**
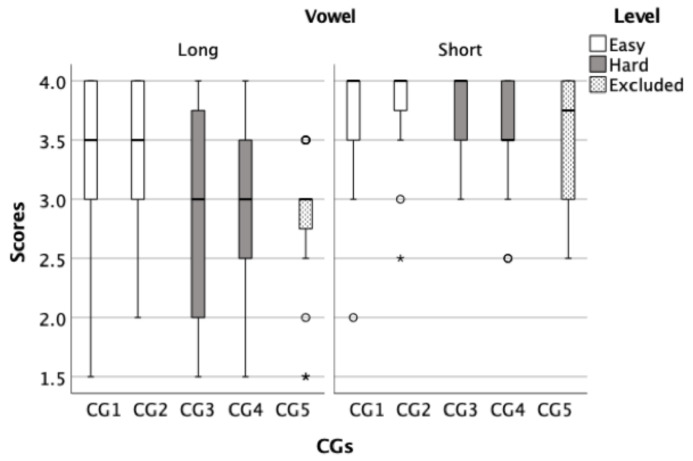
In the box plots, the *Y* axis represents the average correct score for the test and retest trials and the *X* axis represents the developed initial phoneme CGs. The left panel shows the groups with long vowels whereas the right panel shows the groups with short vowels. White boxes represent CGs that was included in the easy list and grey boxes represent the CGs that was included in the hard lists. Dotted boxes represent CGs that were excluded from the final word lists.

**Figure 4:**
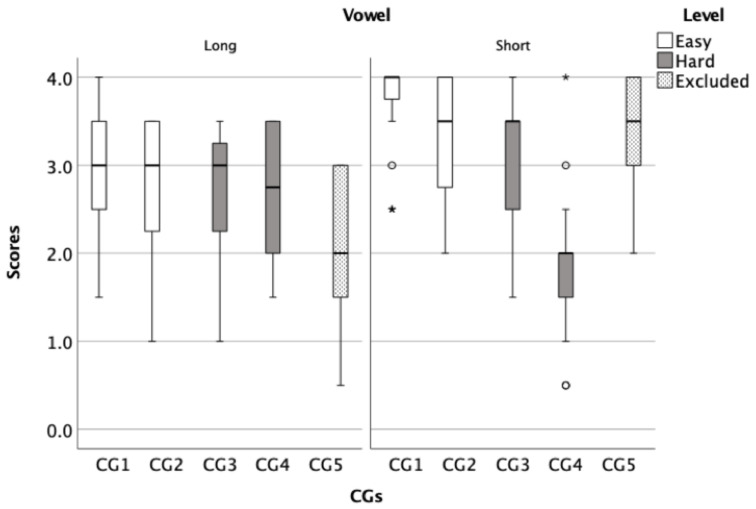
In the box plots, the *Y* axis represents the average correct score for the test and retest trials and the *X* axis represents the CGs (final phonemes). The left panel shows the groups with long vowels whereas the right panel shows the groups with short vowels. White boxes represent CGs that was included in the easy list and grey boxes represent the CGs that was included in the hard lists. Dotted boxes represent CGs that were excluded from the final word lists.

In this phase, data were collected to evaluate the developed lists from phase I, of which there were four (two easy and two hard lists). The level of difficulty of the lists was determined based on the average scores for each CG (Figure 3 and Figure 4). Data were analyzed for individual lists and also by combining the two easier lists and combining the two harder lists producing a total of two lists, an easy list and a hard list. Each list can be used repeatedly by merely changing the order of the words within the list because all words are represented on each run (See Table 2).

##### Participants

Children were recruited via advert at King Abdul-Aziz University in Jeddah, Saudi Arabia. Sixteen children aged between five and eleven years (mean age = 8.33 years) participated in the validation of this phase, nine males and seven females. All children were screened at 20 dB HL using pure-tone audiometry at frequencies 0.5, 1.0, 2.0 and 4.0 kHz. All children passed hearing screening and none were reported to have learning disabilities.

##### Technical Delivery

Experiments were conducted in a quiet room in King Abdul-Aziz University or at the participant’s home. The noise floor was measured using a sound level meter to be equal or less than 40 dBA. The test materials were delivered via a computer running a MatLab script to present stimuli, show response options and record responses. The participants were instructed to select one out of four pictures that visually and orthographically represent the presented word. Each participant was tested individually; the child listened to the stimuli over Sennheiser HD 650 headphones and selected the corresponding picture out of four choices shown on the computer’s screen that visually and orthographically represented the presented word. The lists were presented at four different levels starting at soft level 40 dB SPL, then increasing the volume by 10 dB SPL increments (50, 60, and 70 dB SPL) until the child reached the maximum score. The four different presentation levels were used to map out the psychometric function (performance by level) so that the most appropriate presentation level for future test presentation could be established. This psychometric function could also be used to find a presentation level for test re-test reliability and critical difference calculations for a performance level that was not at the floor or ceiling of the performance range. Each test was run twice before increasing the volume to the next level with a ten-minutes break in between; order of words was altered to minimize learning effect.

## 3. Results

### 3.1. Phase I: Assessment of Speech Materials and Development of Lists

In this phase, the speech materials were designed to assess two distinct skills, discrimination of initial consonant and final consonant of monosyllabic words. Therefore, the test and analysis were conducted in two parts that assessed each skill separately. Three measures were utilized to evaluate the appropriateness and intelligibility of the developed materials. The first measure was the scores of participants in each CG, where scoring was calculated by adding the number of correct words within a CG, then dividing it by the total number of words within a CG producing an average score for each CG. Even though the test was presented at a soft level (50 dB SPL), participants’ scores were overall high with an average of 3.4 points out of 4 for initial phoneme CGs and 2.8 points out of 4 for final phonemes CGs. Scores in the final phoneme component were consistently lower, with the lowest score for the word /bar/. The word /bar/, which means land (particularly referring to desert in Saudi Arabic) is pronounced the same in the colloquial and Modern Standard Arabic and is a very familiar word to children in Saudi Arabia. It is worth noting that such a low score was not caused by the use of Modern Standard Arabic language but potentially due to the difficulty of perception of the acoustic cues for /bar/ when presented at a low level.

The second measure was intraclass correlation coefficient (ICC) to assess the agreement between the participants’ scores within each CG, where responses of all participants for each CG were assessed to evaluate the degree of agreement between participants. We used two-way mixed-effects ICC model with type consistency to assess agreement between subjects’ averaged scores at each CG in the test and retest runs. The ICC showed excellent agreement of 0.94 between the participants in both the initial and final phoneme components, suggesting a significant correlation between participants’ responses in each CGs.

The third measure was the comparison of the average scores of each CG. Two ANOVAs were conducted, one for initial phoneme and one for final phoneme. For both, a repeated measures ANOVA with within-subject factors of Test session (one and two) and Word group (10 CGs) were used. If the Mauchley’s test of Sphericity was significant we used Greenhouse-Geisser corrections. For the initial phoneme, there was no significant effect of Test session, F (1, 22) = 0.04, *p* = 0.84 s, but a significant main effect of Word group, F (4.371, 96.168) = 14.589, *p* < 0.001. The results also show significant interactions between Test session and Word group, F (5.056, 111.233) = 2.981, *p* = 0.014, where participants’ scores for CG2 were significantly higher by an average increase of 0.4 points in the retest session. For the final phoneme contrast there was no significant main effect of Test session F (1, 18) = 0.945, *p* = 0.344 but there was a significant main effect of Word group, F (9, 162) = 14.608, *p* < 0.001. Pairwise comparison generally showed that the mean scores for the CGs for the long vowel words were significantly different from mean scores for the CGs for the short vowel words, for both initial and final phonemes tests. The results also show significant interactions between Test session and Word Group, F (9, 96.966) = 7.580, *p* < 0.001, where participants’ scores at CG2, CG4 in the long vowel CGs and CG2 in the short vowel CGs were significantly different in the retest session by an average change of −1.2, 0.85, and 0.6 points, respectively.

To show the average score of CGs, box plots for both the initial (Figure 3) and final phoneme components (Figure 4) were created to illustrate the overall performance of the children. These figures were used to analyse the similarities and differences between CGs within each test to inform the development of the final word lists. After analysing the CGs, the CGs that had lower average scores were considered difficult and were selected for a harder list. Then, CGs were sorted based on its level of difficulty from least confusing to most confusing, subsequently four lists were developed, two of which were easy and two were hard. For example, CG1 in Figure 3 consisted of the words /naːr/, /daːr/, / tˤaːr/, and /ħaːr/ had an average score of 3.38 (out of 4) while CG5 in the same panel consisted of /baːb/, /ðaːb/, /ʃaːb/, and /ɣaːb/ had and average score of 2.85 (out of 4), indicating that the CG1 is easier (less confusing) than the CG5.

### 3.2. Phase II: Validation of the Developed Lists: Lists Equivalency Analysis

Using the lists (two easy and two hard) that were developed in phase I, an experiment was conducted to evaluate the difficulty and equivalency across lists. Since the difference between the lists was most apparent at lower presentation levels and ceiling effects were observed in the higher presentation levels (Table 3), only the data that were collected at 40 dB SPL were used to evaluate the list equivalency and produce the final form of the lists. A repeated-measure ANOVA was performed with a within-subject factor Test Sessions (test and re-test) and Lists (four lists) and Sphericity was assumed.

**Figure 5:**
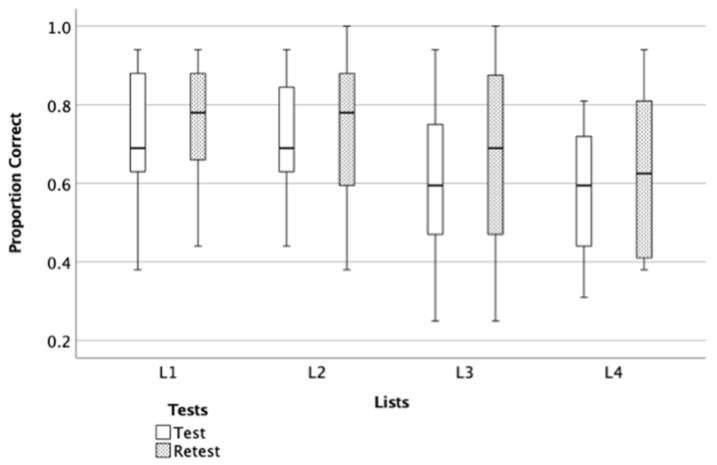
In the box plots, the *Y* axis represents the proportion correct score and the *X* axis represents the developed four lists. The boundary of the box closest to zero indicates the 25th percentile, a black line within the box marks the median, and the boundary of the box farthest from zero indicates the 75th percentile. Whiskers above and below the box indicate the 10th and 90th percentiles.

The results indicate a significant main effect of List, F (3, 13) = 12.1, *p* < 0.001 and a significant main effect of Test Sessions, F (1, 15) = 5.54, *p* = 0.03, suggesting the existence of different level of difficulty within lists and performance in retest session. The results show no significant interaction between the effects of Lists and Test Sessions on scores, F (3, 13) = 0.96, *p* = 0.44. Post hoc comparisons using the Tukey HSD test indicated that the mean scores for List 4 was significantly lower than the mean scores of List 1 (*p* < 0.001) and List 2 (*p* < 0.001). In addition, the mean score of List 3 was significantly lower than the mean scores of List 2 (*p* = 0.03) and (*p* = 0.05) List 1 (Figure 5). The results indicate that List 1 and List 2 were easier than List 3 List 4. In addition, participants’ scores increased by an average of 0.05 points in retest session.

### 3.3. Critical Difference

When the initial critical difference calculations were conducted with the four lists, reliability was not satisfactory. To address this, lists were combined to increase the number of items in a list. The two easy lists were merged into one list of 32 items and the two hard lists were also merged into one list of 32 items. This improved reliability of the test by increasing the number of items within lists.

A within-subject sω was calculated (as calculated in the original CAPT) to derive the 95% confidence interval of the score for an individual [28]. The quantity sω is the square root of the mean group variance (mean across individuals of the variance calculated for each individual). An individual’s observed score is expected to lie within ±1.96 sω of their true score (for 95% of observations; the CI). The critical difference is calculated as √2 × 1.96 sω. If scores obtained on two different occasions differ by √2 × 1.96 sω or more, then they differ significantly at *p* < 0.05.

**Figure 6:**
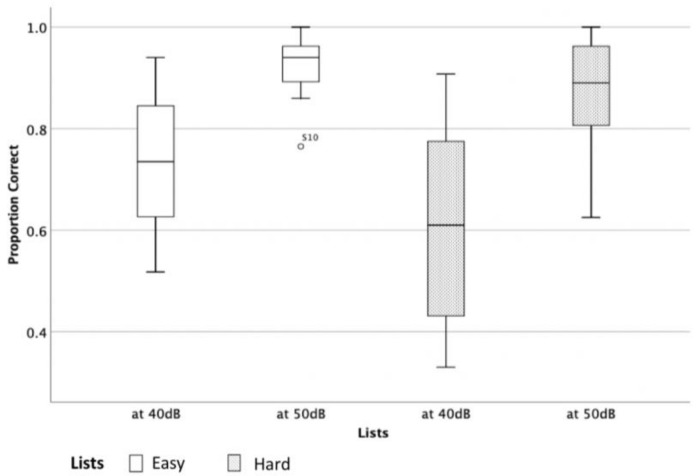
Proportion correct scores for the easy and hard lists at 40 and 50 dB SPL.

The critical difference was calculated for both levels 40- and 50-dB SPL. However, it was decided to present the 50 dB SPL calculations as 50 dB SPL was determined to be the preferred presentation level since 40 dB SPL is considered soft speech and could be affected by variation of hearing thresholds and noise floor as observed by the participants’ performance (see Figure 6).

The mean critical difference at 50 dB SPL for the easy list was 18% and the hard list was 12%. Table 4 shows how the critical difference varies across the performance range at 50 dB SPL and what the critical difference is for an individual score out of 32. For example, a child scored 88% on the easy list one occasion and 77% in the second. This would not be considered as a significant change in performance since the score falls between 69% and 100%. However, if the child scored 66% on the second occasion, this would be viewed as a significant decrease in performance.

### 3.4. Test–Retest Reliability and Age Effect

**Figure 7:**
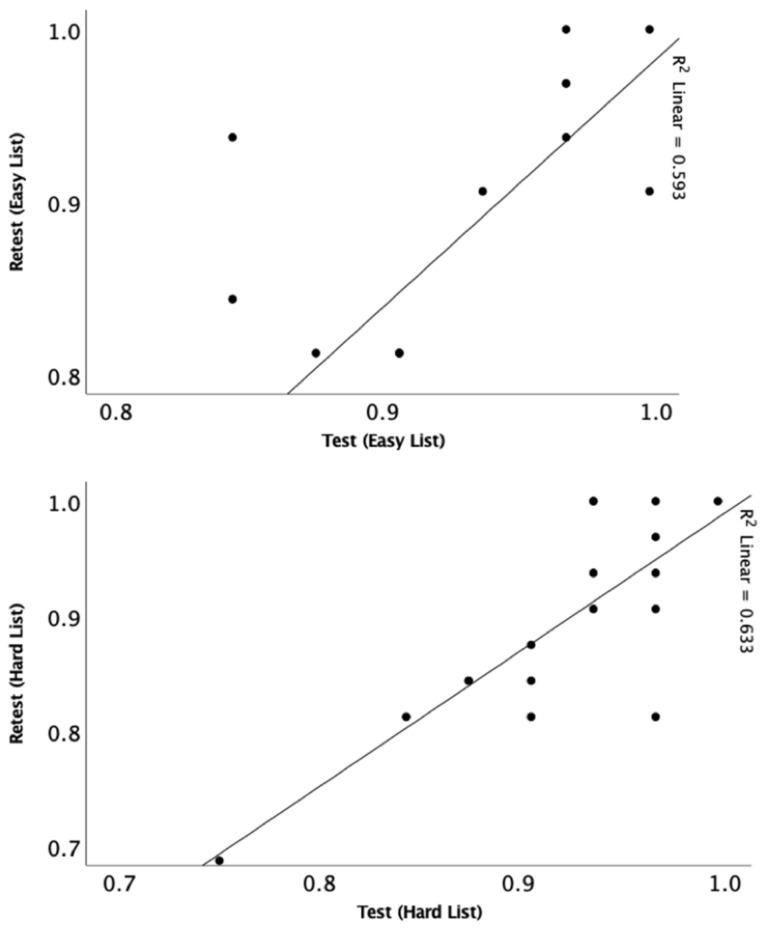
Depicts the relationship between test and retest 50 dB SPL at easy (**top**) and hard list (**bottom**).

**Figure 8:**
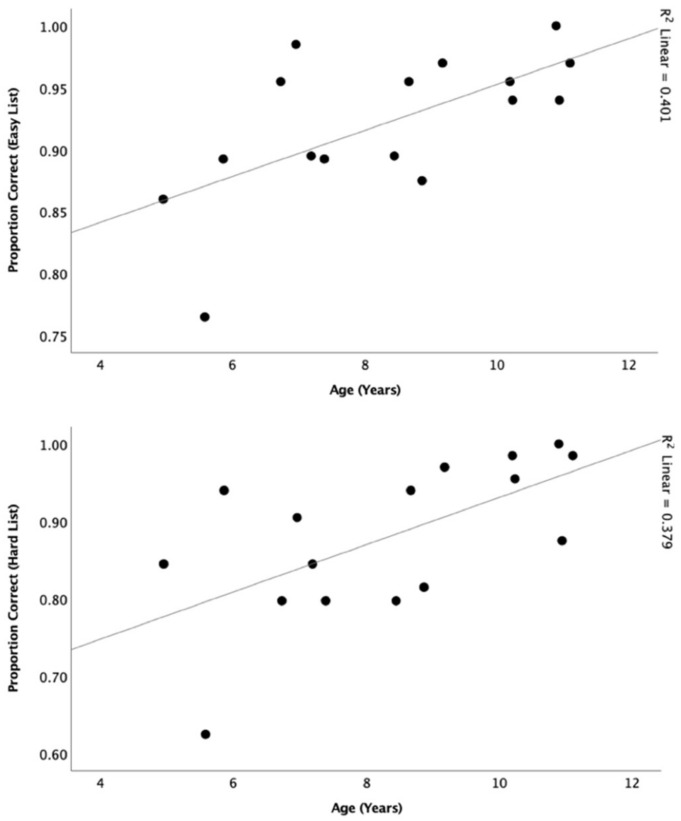
Depicts the relationship between age and proportion correct scores at easy (**top**) and hard (**bottom**) lists at 50 dB SPL.

Pearson correlation was conducted to assess test–retest reliability and showed significant correlations at 50-dB SPL for easy (r = 0.77, *p* < 0.001) and hard (r = 0.79, *p* < 0.001) lists (Figure 7). Pearson correlations were also conducted to determine the relationship between participants’ performance and age at 50 dB SPL (*n* = 16) (Figure 8). There was a significant relationship for both the easy list (r = 0.63, *p* = 0.01) and hard list (r = 0.62, *p* = 0.01).

## 4. Discussion

A new Arabic monosyllabic closed-set consonant-discrimination test, for use with Arabic-speaking children over the ages of five years has been developed. The main motivation for this research was the lack of materials that can be used with this population to assess their consonant perception skills and monitor changes over time or after an intervention. The new test is an adaptation of the CAPT into Modern Standard Arabic.

Selecting the format of the speech test is key to ensure that the measurement is assessing the desired function in a reliable way [29,30]. We therefore decided to use a closed-set format because this makes the task a discrimination task, which is easier for younger children, not reliant upon a child’s ability to produce sounds, and shows how different phonemes are confused. This analytical information can be used to guide fitting to optimise delivery of speech information. The mode of delivery of this test was auditory-only in a quiet environment following the standard approach used for the CAPT [21]. We chose to adapt the CAPT because it has been shown to be a reliable outcome measure for assessing speech perception in young children and is sensitive to hearing aid gain settings [22] and differences in the audiogram [24].

To develop this word discrimination test, the word familiarity and language level was determined to be aligned with recommendations from other authors [29,30]. This was done by presenting the selected Arabic words in Modern Standard Arabic to a group of primary-school Saudi Arabian children to assess the familiarity and appropriateness of the words and the clarity of the corresponding pictures. The scores of the children in the development stage were generally very high suggesting that the pictures and words were intelligible for the majority of normal hearing participants. This part of the study was conducted in the UK at the KFA, a Saudi-funded primary school. The children at this school who partook in this experiment were from Saudi Arabia and were exposed to the same type of Modern Standard Arabic as those children who participated in the final phase of the study that was conducted in Saudi Arabia. The only difference between these two groups was the fact that most of the children who participated in the study that was conducted in the UK were bilingual while most of the children who participated in the study conducted in Saudi Arabia were monolingual. Such a difference is not expected to be an issue since all the children were native Arabic speakers who were equally exposed to Modern Standard Arabic.

The above scenario illustrates the benefits of using the formal form of the language with school-aged children when dealing with diglossic languages such as Arabic, Modern Greek, Swiss German, and Haitian Creole. This study showed that despite the existence of various number of dialects within the Saudi-Arabic language, children with different backgrounds were familiar with the materials that were presented in a closed-set format in the formal form of the language and achieved high scores in this test. This may also indicate that the test can be used with Arabic-speaking children in non-Arabic-speaking countries. For example, it has been reported that Arabic is the fastest growing language in the United States [31]. In addition, providing such tests can minimize the reported lack of materials for assessing speech in children who lives in the UK but speaks languages other than English [32]. In large cities, it has been reported that the proportion of children who have English as not their first language can be higher than those who have English as the family language [33].

The final version of the A-CAPT differs slightly from the original CAPT [21]. The A-CAPT consisted of two lists, one was considered ‘easy’ and one ‘hard’, where each list consisted of eight CGs producing a total of sixteen CGs. The original CAPT [21] has only one stimulus set consisting of twelve CGs (48 items) which are presented in multiple lists comprised of different presentation orders of the items. For each of list in the A-CAPT the measures were shown to have strong agreement between the test and retest sessions with Pearson correlation values of 0.77 and 0.95 for the easy and hard lists, respectively; these values were similar to the reported value (0.83) in the original CAPT.

The effect of age is a critical element to evaluate when validating a speech perception measure [29,30]. There was a significant correlation between the scores on the test and the age of the participants, with older children performing better than younger children. Such trend was expected as older children with normal hearing are reported to have better speech discrimination skills and advanced spectral resolution maturity compared to younger children [34,35]. Age accounted for approximately 40% of the variance for both the easy and hard lists. The relationship did not reach a stronger level possibly due to the small sample size (*n* = 16) used in this experiment. However, this relationship was stronger than the reported in the original CAPT [21], which only accounted for 15% of the variance, which was explained by the limited spread of ages of participants used in the CAPT validation [21].

The critical differences for the two lists can be used to determine whether or not changes in performance of an individual child are significant. The critical difference values reported for the CAPT was 13.7%, which is similar to the critical values that we calculated for the hard list (12%). For the easy list, the average critical difference was larger (18%). These critical difference values indicate that the harder list is a better discriminator when comparing an individual child’s performance in two different conditions

Finally, limitations of the test and study should be noted. Firstly, the sample size in the development of material phase (*n* = 26) and lists’ evaluation phase (*n* = 16) of this study was smaller than the original CAPT (*n* = 55). In addition, the range of ages was wider, where the range of ages in the CAPT was 4–8 years and in the A-CAPT it was 5–11 years. Further work to determine the age ranges for the final version of the A-CAPT needs to be conducted with a larger number of participants at each year. Secondly, the critical differences are larger than would be desired but in a similar region to the CAPT [21]. This is typical for measures used with young children. It does mean that in an ideal clinical situation that two lists would be conducted to improve the confidence in the scores or at least a short practice list is needed prior to running the actual test. As with all speech measures for children, it means that on the individual level small changes in performance will not be detected. The critical differences were larger at the lower level (40 dB SPL compared to 50 dB SPL), and this is again as expected because the children were being tested at a lower point on the psychometric function closer to their hearing threshold, where greater variability is typically observed. Third, this test is conducted in Modern Standard Arabic, which is not the everyday mother-tongue spoken language by Arabic-speaking children, but rather a formal form used in media and at school. It was selected because the variation in dialects for Saudi Arabic is vast and the Modern Standard Arabic can be considered as a common ground that everyone is exposed to on daily basis, including the selected age group of children.

## 5. Conclusions

This A-CAPT has been developed to assess consonant perception in Arabic-speaking children aged 5 years and older. The test consists of an easy list and a hard list, which were validated with normal-hearing children (aged 5 to 11 years). Test–retest reliability was high for both the easy and hard lists. Overall, children’s performance improved with increasing age. Just like the CAPT [21] from which it was adapted, the A-CAPT uses a wide range of phonemes in a speech discrimination task that will be helpful when programming hearing devices or planning an intervention.

Although the A-CAPT was developed to replenish the lack of tools that could assess consonant perception in Arabic-speaking children in Saudi Arabia, this test is also relevant and usable for other Arabic-speaking children in other countries where dialect-specific speech tests are not available. The A-CAPT allows the assessment of the discrimination of speech cues and provides frequency-specific information with known reliability; this information is helpful to clinicians to assess auditory perception, verify the benefits of hearing devices, and assess effectiveness of habilitation interventions.

## 6. Future Work

Continuation of this work would be to conduct a large-scale validation of the A-CAPT test with larger sample size that can produce normative data for different age groups. In addition, speech materials for younger children using regional dialects can be developed. A version of the A-CAPT can also be validated with the words presented in background noise. Finally, assessment of the A-CAPT in other Arabic countries can be completed to determine if Standard Arabic can be used more widely.

## Figures and Tables

**Table 1:** Published speech tests validated with Saudi-Arabic children.

Name	Type	Target Age	Dialect
Arabic Lexical Neighborhood Test (LNT) [17]	Open-set word recognition test	5–13 year old children with CI	Colloquial Najdi Arabic
Arabic Matrix Sentence test [25]	Closed-set sentence recognition	12 year old children and above	Modern Standard Arabic
SRT test in Saudi [7]	Open-set	5 year old and above	Modern Standard Arabic

**Table 2:** Two lists were developed, an easy List and a hard List.

A-CAPT Lists			
Easy List		Hard List	
Long Vowel	Short Vowel	Long Vowel	Short Vowel
/suːr/	/jad/	/naːs/	/ħar/
/duːr/	/sad/	/maːs/	/bar/
/nuːr/	/yad/	/daːs/	/jar/
/ħuːr/	/xad/	/baːs/	/mar/
/naːr/	/batˤ/	/tˤiːn/	/ʕam/
/daːr/	/xatˤ/	/liːn/	/fam/
/tˤaːr/	/natˤ/	/tiːn/	/kam/
/ħaːr/	/matˤ/	/diːn/	/dam/
/suːs/	/xatˤ/	/ħaːdʒ/	/bar/
/suːd/	/xas/	/ħaːb/	/bard/
/suːr/	/xal/	/ħaːd/	/batˤ/
/suːq/	/xad/	/ħaːr/	/barq/
/qaːs/	/mawt/	/tˤiːb/	/kab/
/qaːl/	/mawz/	/tˤiːħ/	/kaf/
/qaːm/	/mawdʒ/	/tˤiːn/	/kam/
/qaːd/	/mar/	/tˤiːr/	/kaħ/

**Table 3:** The mean of participants’ scores (shown in proportions) at 40, 50, 60, and 70 dB SPL.

dB SPL /Lists	L1	L2	L3	L4
**40**	0.74	0.71	0.66	0.56
**50**	0.93	0.90	0.89	0.86
**60**	0.94	0.93	0.95	0.83
**70**	0.92	0.95	0.92	0.89

**Table 4:** Critical differences (CD) for easy and hard lists expressed in percentages (95% confidence interval).

Easy List at 50 dB SPL	CD −+18%		Hard List at 50 dB SPL	CD −+12%	
Scores (Out of 32, %)	Lower Boundary	Upper Boundary	Scores (Out of 32, %)	Lower Boundary	Upper Boundary
100	82	100	100	88	100
97	79	100	97	85	100
94	76	100	94	82	100
91	72	100	91	79	100
88	69	100	88	76	100
84	66	100	84	73	100
81	63	99	81	69	93
78	60	96	78	66	90
75	57	93	75	63	87
72	54	90	72	60	84
69	51	87	69	57	81
69	51	87	69	57	81
66	47	84	66	54	77
63	44	81	63	51	74
59	41	78	59	48	71
56	38	74	56	44	68
53	35	71	53	41	65
50	32	68	50	38	62
47	29	65	47	35	59
44	26	62	44	32	56
41	22	59	41	29	52
38	19	56	38	26	49
34	16	53	34	23	46
31	13	49	31	19	43
28	10	46	28	16	40

## Data Availability

Not applicable.
